# Monomeric annexin A2 is an oxygen-regulated toll-like receptor 2 ligand and adjuvant

**DOI:** 10.1186/s40425-016-0112-6

**Published:** 2016-02-16

**Authors:** Brian M. Andersen, Junzhe Xia, Alan L. Epstein, John R. Ohlfest, Wei Chen, Bruce R. Blazar, Christopher A. Pennell, Michael R. Olin

**Affiliations:** Department of Neurology, New York-Presbyterian/Weill Cornell Medical Center, 525 Ease 68th St, room F-610, New York, NY 10065 USA; Department of Neurosurgery, Hospital Number 1 of China Medical University, Shenyang, China; Department of Pathology, University of Southern California, Los Angeles, CA USA; Department of Pediatrics and the Masonic Cancer Center, University of Minnesota, Minneapolis, MN 55455 USA; Department of Pediatrics and the Masonic Cancer Center, 8366A, 420 Delaware St SE University of Minnesota, Minneapolis, MN 55455 USA; Department of Laboratory Medicine and Pathology, University of Minnesota, Minneapolis, MN 55455 USA; Department of Pediatrics and the Masonic Cancer Center, 3-136 CCRB, 2231 6th St SE, University of Minnesota, Minneapolis, MN 55455 USA

**Keywords:** Adjuvant, Annexin, Glioblastoma, TLR2, Vaccine

## Abstract

**Background:**

Annexin A2 (ANXA2) is a pleiotropic, calcium-dependent, phospholipid-binding protein with a broad tissue distribution. It can be intracellular, membrane-bound, or secreted, and it exists as a monomer or heterotetramer. The secreted ANXA2 heterotetramer signals human and murine macrophages to produce IL-1, IL-6, and TNF-α through TLR4/MyD88- and TRIF-dependent pathways.

**Methods:**

GL261 glioma cells were cultured in 5 % or 20 % O_2_. Monomeric ANXA2 (ANXA2m) was identified as a TLR2-binding protein enriched in 5 % O_2_ by mass spectrometry. Purified ANXA2m and ANXA2-derived peptides were added to TLR2-expressing reporter cells and immature dendritic cells (DCs) cells in vitro. ANXA2m was then mixed with chicken ovalbumin (OVA) for vaccination of *TLR2*^*+/+*^ and *TLR2*^*−/−*^ mice for subsequent quantification of antigen-specific CD8^+^ T cell responses. The TLR2-binding region of ANXA2m was determined using various peptides derived from the ANXA2 amino terminus on TLR2 reporter cells and in vaccinated mice.

**Results:**

ANXA2m is overexpressed by murine glioblastoma GL261 cells grown under 5 % O_2_, but not atmospheric 20 % O_2_, and acts as an adjuvant by inducing murine and human DC maturation through TLR2. ANXA2m upregulates CD80 and CD86 expression, enhances antigen cross-presentation, and induces the secretion of IL-12p70, TNF-α, and IFN-γ. The amino-terminal 15 amino acids of ANXA2m are necessary and sufficient for TLR2 binding and DC activation.

**Conclusion:**

This novel finding adds to the known functions of ANXA2 and suggests ways to exploit it as a vaccine adjuvant. ANXA2-antigen fusion peptides could be developed for patients as “off-the-shelf” agents containing common tumor antigens. Alternatively, they could be “personalized” and synthesized after tumor sequencing to identify immunogenic tumor-specific neo-antigens. As the amino terminal 15 amino acids of ANXA2 are required to stimulate TLR2 activity, a fusion peptide could be as short as 30 amino acids if one or two CD8 T cell epitopes are fused to the ANXA2 amino terminal portion. Future work will address the efficacy of ANXA2 peptide fusions alone and in combination with established TLR agonists to induce synergy in preclinical models of glioma as observed in other vaccines.

## Background

Annexin A2 (ANXA2)^4^ is a broadly expressed member of the annexin family of calcium-dependent, anionic phospholipid-binding proteins. Annexins have homologous core and carboxyl domains but variable amino (N)-terminal regions with diverse binding and functional properties. These variable N-terminal 30 amino acid regions, called head regions, distinguish the family members [1]. ANXA2 was first reported as an intracellular membrane-associated substrate of the pp60v-src oncoprotein in transformed chicken embryonic fibroblasts [2]. ANXA2 is now known to be expressed by many cell types in the nucleus, cytoplasm, membrane, or extracellularly. Its many functions include membrane organization and dynamics during endocytosis and exocytosis, gene regulation, ion channel formation, fibrinolysis, and cellular transformation [3, 4]. The subcellular localization, post-translational modifications, and different binding partners of ANXA2 dictate its diverse functions.

A frequent binding partner of ANXA2 is the plasminogen receptor protein S100A10 [5]. Upon binding to calcium, a conformational change occurs in ANXA2m that exposes a hydrophobic residue in its N terminus, allowing ANXA2m to bind a S100A10 dimer. This dimer bridges two ANXA2m to form a heterotetramer (ANXA2t) [4]. ANXA2t binds anionic phospholipids with high affinity and correspondingly often localizes to the plasma membrane. ANXA2t can also be released from cells in exosomes (after Tyr-23 phosphorylation), via SNARE-mediated fusion with the plasma membrane, and through transmembrane channels (when chaperoned by PtdIns [4, 5] P2) [6–8]. Extracellular ANXA2t functions immunologically by inducing macrophages to release IL-1, IL-6, and TNF-α through TLR4/MyD88- and TRIF-dependent pathways [9].

Here we report for the first time that extracellular ANXA2m also functions immunologically, and that the monomeric and tetrameric forms of ANXA2 signal through different pathways. We find that ANXA2m, when released by cells, binds TLR2 and induces the differentiation of murine and human antigen presenting cells (APCs). ANXA2m induces the expression of CD80 and CD86, the secretion of IL-12, INF-γ, and TNF-α, and enhances the cross-priming of murine and human antigen-specific CD8^+^ T cells. All of these activities map to the unique N-terminus of ANXA2m. These data, combined with our observation that ANXA2m is over-expressed by murine GL261 glioblastoma cells grown under low (5 %) but not atmospheric (20 %) O_2_ levels, suggest ANXA2m is a novel danger-associated molecular pattern (DAMP) that contributes to the increased efficacy of tumor vaccines derived from GL261 cells grown under 5 % O_2_ [10].

## Results and discussion

### ANXA2m is an O_2_-regulated protein that binds TLR2

We reported that decreasing O_2_ from the typical tissue culture level of 20 to 5 % enhanced the adjuvant activity of lysates from murine GL261 glioma cells in a glioblastoma vaccination model [10]. This led us to hypothesize that 5 % O_2_ enriched for the expression of DAMPs by GL261 cells that, in turn, activated APCs by binding to pattern recognition receptors. Because the pattern recognition receptor TLR2 was implicated in inflammation following hypoxia in the brain [11], we further hypothesized that TLR2 was required for recognizing O_2_-regulated putative DAMP expression in GL261 cells.

**Fig. 1:**
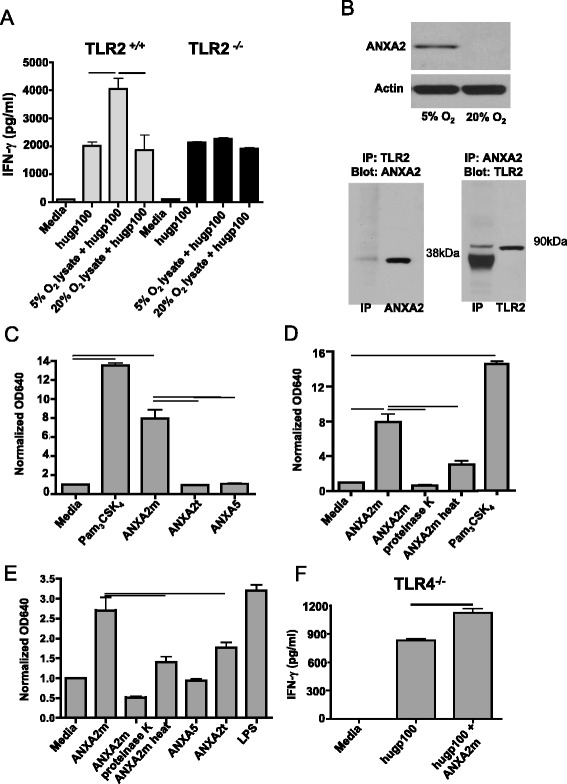
ANXA2 is enriched in physiologic oxygen, the monomeric form of which is a TLR2 agonist. **a** Splenocytes from B6 (*TLR2* ^*+/+*^) and *TLR2* ^*−/−*^ mice were pulsed with the human gp100_25–33_ peptide, with or without lysates from GL261 cells grown under 5 % or 20 % O_2_. Addition of purified pmel-1 CD8 T cells resulted in INF-γ secretion, which was quantified after 48 h by flow cytometry. The experiment was repeated three times with similar results (the average INF-γ median fluorescence intensities [MFIs] for the three experiments were 1,868 and 4,057 for splenocytes stimulated with lysates from GL261 cells grown under 5 % or 20 % O_2,_ respectively). Lines indicate *p* <0.05 by unpaired t-test. **b** (*top*) Protein in GL261 lysate was separated by SDS-PAGE and immunoblotted for murine ANXA2. (*bottom*) ANXA2 and TLR2 in GL261 lysates coimmunoprecipitate as shown by immunoblot. Recombinant murine ANXA2 (38 kDa) and TLR2 (90 kDa) were used as controls. **c** & **d** Compounds (6.9 μM) were pulsed onto human TLR2-expressing HEK-Blue cells, and secreted alkaline phosphatase was measured. “Proteinase K” and “heat” respectively indicate pre-treatment with the enzyme or incubation of the protein at 56 ° C for 30 min prior to addition to reporter cells. Y axes represent colorimetric quantification of alkaline phosphatase activity, measuring absorbance at 640 nm and normalized to media controls. The experiment was repeated three times with similar results. The mean absorbances at 640 nm for these three experiments were 7.2 and 1.4 for ANXA2m and ANXA2t, respectively. **e** Same as c and d only human TLR4-expressing HEK-Blue cells were used. The experiment was repeated three times with similar results. The mean absorbances at 640 nm for these three experiments were 2.7 and 1.7 for ANXA2m and ANXA2t, respectively. **f** Same as (a) except that splenocytes from *TLR4* ^*−/−*^ mice were pulsed with the human gp100_25–33_ peptide with or without human ANXA2m. The experiment was repeated three times with similar results (the average INF-γ MFIs for the three experiments were 1,126 and 831 for peptide-pulsed splenocytes with or without human ANXA2m, respectively). Error bars are ± SEM, and lines show *p* <0.01 by unpaired t-test

To test these hypotheses, we pulsed splenocytes from wildtype B6 or *TLR2*^*−/−*^ mice with the human melanocyte antigen-derived gp100_25–33_ peptide, with or without lysates from GL261 cells grown under 5 % or 20 % O_2_. The splenocytes were then compared for their abilities to activate purified CD8^+^ T cells bearing the transgenic pmel-1 Vα1/Vβ13 TCR specific for human gp100_25–33_ presented by the MHC class I molecule H-2D^b^ [12]. We found antigen-specific CD8^+^ T cell activation, as measured by IFN-γ expression, required both TLR2 and lysate from GL261 cells cultured under 5 % O_2_; lysates from GL261 cells grown under 20 % O_2_ had no stimulatory effect on either *TLR2* sufficient or deficient splenocytes (Fig. 1a). These data suggest TLR2 recognizes a DAMP expressed by GL261 cells cultured under 5 % O_2_.

To identify the putative DAMP(s), we screened lysates from GL261 cells cultured under 5 % O_2_ for TLR2-binding molecules using immunoprecipitation. Subsequent mass spectrometry revealed ANXA2 was enriched in the TLR2-binding fraction (data not shown). Immunoblot analysis confirmed that ANXA2 was overexpressed in GL261 cells grown under 5 % O_2_ relative to 20 % O_2_ (Fig. 1b, top). Binding of ANXA2 and TLR2 was corroborated by co-immunoprecipitation (Fig. 1b, bottom; the <90 kDa bands in the anti-TLR2 immunoblot correspond to lower molecular weight TLR2 isoforms) [13]. Together these data reveal ANXA2 is an O_2_-regulated protein that binds TLR2.

We next asked which form of ANXA2 bound TLR2, and if binding resulted in signal transduction. ANXA2m and ANXA2t were incubated in vitro with HEK-Blue reporter cells expressing human TLR2. Secretion of alkaline phosphatase by these reporter cells was induced by human ANXA2m, and the TLR2-signaling positive control Pam_3_CSK_4_ (a synthetic analogue of triacylated lipopeptide), but not by bovine ANXA2t (Fig. 1c). Heat denaturation or enzymatic degradation of human ANXA2m abolished signaling, showing the TLR2 binding moiety was proteinaceous and not a contaminating lipid moiety (Fig. 1d). Human ANXA2m did not induce alkaline phosphatase secretion by the HEK-Blue human TLR7 reporter cell line, showing the specificity of ANXA2m binding to TLR2 relative to TLR7 (data not shown).

Because ANXA2t binds and signals through TLR4 [9], we tested whether ANXA2m could do the same. We found that human ANXA2m was significantly superior to an equimolar amount of bovine ANXA2t at activating the HEK-Blue human TLR4 reporter cell line (Fig. 1e). Although ANXA2m is of human origin, and ANXA2t is bovine, the two ANXA2 proteins have identical head regions and share 99 % homology overall. It is therefore unlikely the enhanced signaling through human TLR4 of the monomer relative to the tetramer is due to species differences.

To determine if ANXA2m could enhance an antigen-specific T cell response, purified CD8^+^ T cells bearing the pmel-1 TCR were added to cultures of APCs from *TLR4*^*−/−*^ mice pulsed with the gp100_25–33_ peptide with or without human ANXA2m. ANXA2m significantly enhanced antigen-specific activation of pmel-1 CD8^+^ T cells, as measured by IFN-γ expression compared to the peptide alone (Fig. 1f). Together these data demonstrate ANXA2m, and not ANXA2t, binds and signals through TLR2, and the ANXA2m adjuvant effect does not require TLR4 or TLR7.

### ANXA2m enhances murine and human antigen-specific CD8^+^ T cell responses

Unlocking the cross-priming potential of the DC is a cardinal requirement of tumor cell vaccines. Patient-derived immune suppression or poor immunogenicity of cell lysates can limit the impact of co-delivered synthetic adjuvants thereby diminishing DC activation and cross-priming. Therefore, we next asked whether the addition of ANXA2m to DC cultures could increase cross-priming.

**Fig. 2:**
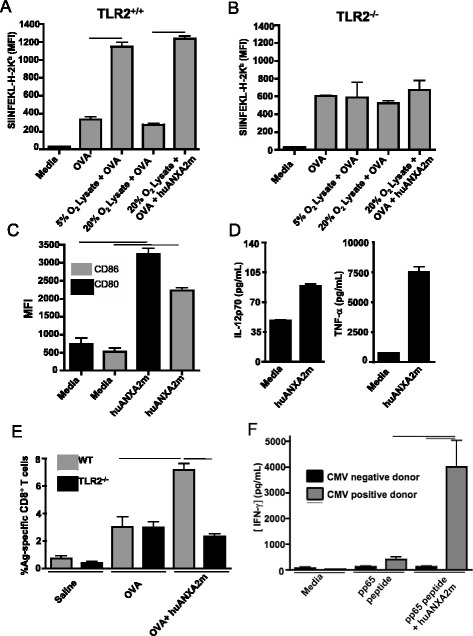
ANXA2m enhances dendritic cell maturation and cross-priming in a TLR2-dependent manner. **a** Splenocytes from *TLR2* ^*+/+*^ mice were pulsed with the OVA_248–274_ peptide SIIFNEKL. SIINFEKL cross-presentation was detected 24 h later by flow cytometry using the 25-D1.16 antibody (specific for the SIINFEKL/H-2K^b^ complex). Data represent the median fluorescence intensity of cells within a CD11c^+^MHCII^+^ gate. Bars signify *p* <0.002 by unpaired t-test. The experiment was repeated three times with similar results. The average MFIs for the three experiments were 1,236 and 274 for peptide-pulsed CD11c^+^MHCII^+^ splenocytes with or without human ANXA2m, respectively. **b** Same experimental design as (**a**) except the splenocytes were from *TLR2* ^*−/−*^ mice. In all three replicates there were no significant differences among the treatment groups. **c** BMDC cell cultures were pulsed with 2.5 μg/ml human ANXA2m or medium. Cells were harvested and stained after 2 days. Graphs indicate the MFI for the CD11c^+^MHCII^hi^ gated population. The average MFIs from three replicate experiments were 12,736 and 7,577 for CD80 and CD86, respectively. **d** Supernatants from (**c**) were collected and assayed using a cytometric bead array. The averages from the three replicate experiments were 106 and 7,529 pg/ml for IL12p70 and TNFα, respectively. Error bars are ± SEM, and lines indicate *P* <0.01 by unpaired t-test. **e** Naïve mice were vaccinated with four sequential daily injections of OVA (days 1–4), with or without recombinant human ANXA2m and boosted once on day 7. Staining of peripheral blood was performed on day 8. Graph represents the percentage of peripheral blood cells staining positive for the SIINFEKL/K^b^ dextramer within the CD8α^+^ lymphocyte gate. The experiment was replicated twice with similar results with n = 4 per group (the average frequencies of dextramer-binding CD8α + cells were 7.17 and 2.3 % in the wildtype and *TLR2* ^*−/−*^ mice, respectively). Bars denote *p* <0.05 by unpaired t-test. **f** Monocyte-derived dendritic cells from CMV seropositive and seronegative HLA-A0201^+^ donors were pulsed with the pp65_495–503_ peptide, with or without human ANXA2m. After a day cells were washed and autologous PBMCs were added for an additional 2 days. Soluble INF-γ was measured by bead array. The experiment was repeated once with similar results. The averages of the two experiments were 4003 and 114 pg/ml IFN-γ for pulsed PBMCs from CMV seropositive and seronegative individuals, respectively, and 399 pg/ml for unpulsed PBMCs from a CMV seropositive individual. Error bars are ± SEM, and lines show *p* <0.01 by unpaired t-test

To address this question, splenocytes from B6 or *TLR2*^*−/−*^ mice were incubated with or without human ANXA2m and pulsed with both OVA and lysate from GL261 cells cultured in atmospheric (20 %) O_2_. Mouse and human ANXA2 proteins differ by one amino acid residue in their head regions, and share 97 % sequence homology overall. Antigen presentation was measured by flow cytometry using the monoclonal antibody 25-D1.16 (which recognizes the OVA-derived peptide SIINFEKL bound to H-2K^b^) [14]. The addition of human ANXA2m to 20 % O_2_ derived lysates plus OVA significantly increased surface levels of SIINFEKL/H-2K^b^ complexes on splenic DCs (defined as being CD11c^+^, I-A^b+^) compared to the 20 % O_2_ lysates plus OVA (Fig. 2a). As expected, splenic DCs from *TLR2*^*−/−*^ mice did not show enhanced levels of SIINFEKL/H-2K^b^ complexes following stimulation with lysates from 5 % O_2_ or human ANXA2m added to 20 % O_2_ (Fig. 2b). Moreover, human ANXA2m induced robust maturation of B6 wildtype BMDCs as evidenced by a significant increase in the surface expression of the co-stimulatory molecules CD80 and CD86, and secretion of cytokines IL-12p70 and TNF-α (Figs. 2c and d).

To determine if ligation of TLR2 by human ANXA2m enhanced an antigen-specific immune response in vivo, we used a CD8^+^ T-cell priming model that rapidly expands antigen-specific CD8^+^ T cell frequencies [15]. B6 and *TLR*^*−/−*^ mice immunized with OVA plus human ANXA2m were bled and the frequencies of CD8^+^ T cells specific for the OVA-derived peptide, SIINFEKL, presented by H-2K^b^, were determined by flow cytometry. Human ANXA2m significantly (*P* <0.001) increased the frequency of antigen-specific peripheral blood CD8^+^ T cells in wildtype but not *TLR2*^*−/−*^ mice (Fig. 2e). Together these data show human ANXA2m can signal through mouse TLR2 to enhance an antigen-specific immune response.

To determine the effect of human ANXA2m on antigen-specific human CD8^+^ T cell responses in vitro, we pulsed human monocyte-derived DCs from cytomegalovirus (CMV)-positive and -negative donors with a maturation cocktail and the pp65 CMV peptide in the presence or absence of human ANXA2m [16, 17]. The subsequent addition of patient-matched PBMCs led to a ten-fold increase in IFN-γ secretion in CMV seropositive samples when ANXA2m was present. This was an antigen-specific response as PMBCs from CMV seronegative individuals did not secrete IFN-γ in response to pp65 CMV plus ANXA2M (Fig. 2f). These data reveal ANXA2m enhances antigen-specific mouse and human CD8^+^ T cell responses, and suggest human ANXA2m can augment cross-priming in humans in vivo.

### The N-terminal 15 amino acids of ANXA2m are necessary and sufficient for TLR2 binding and signaling

**Fig. 3:**
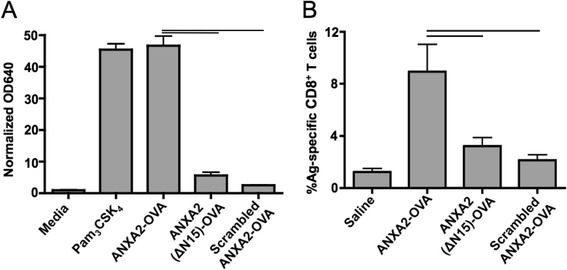
The N terminus of ANXA2 is necessary and sufficient for TLR2 signaling and cross-priming. **a** Peptides derived from the N-terminus of ANXA2 (ANXA2-OVA, the first 36 N-terminal aa from ANXA2 fused to SIINFEKL; ANXA2(ΔN15)-OVA, ANXA2 N terminal residues 16–36 fused to SIINFEKL; scrambled ANXA2-OVA, the first 36 N-terminal residues from ANXA2 randomly ordered and fused to SIINFEKL) were pulsed onto HEK-Blue human TLR2 cells and secreted alkaline phosphatase was measured. The average absorbances at 640 nm from three replicate experiments were 46.7, 5.6, and 2.5 for ANXA2-OVA, ANXA2(ΔN15)-OVA, and scrambled ANXA2-OVA, respectively. **b** Naïve wildtype mice were vaccinated with the sequential daily injection protocol as described in Fig. 2, with OVA plus or minus ANXA2-OVA fusion peptide and derivatives. From three replicate experiments, the average frequencies of dextramer-binding CD8α + cells were 8.6 %, 3.2 % or 2.1 % from mice respectively immunized with ANXA2-OVA, ANXA2(ΔN15)-OVA, or scrambled ANXA2-OVA. Error bars are ± SEM, and lines represent an unpaired t-test *p* <0.05

Members of the annexin protein family have variable 30 aa N-terminal head regions with different sites for post-translational modifications and bind different proteins [1, 3, 4]. As such, they confer unique functions to each annexin. The 15 N-terminal amino acids of ANXA2 bind to the S100A10 protein to form the ANXA2t [4]. Since ANXA2t is unable to activate TLR2, we hypothesized that the N-terminus of ANXA2 was sufficient for signaling through TLR2, and that the first 15 N terminal amino acids were required for TLR2 activity. To test these hypotheses, we incubated HEK-Blue TLR2 reporter cells with peptides containing the first N-terminal 36 amino acid residues of mouse ANXA2 fused to SIIFNEKL (ANXA2-OVA), mouse ANXA2 N-terminal residues 16–36 fused to SIINFEKL (ANXA2ΔΝ15-OVA), or a control peptide containing the first N-terminal 36 aa residues of ANXA2, but with a scrambled order relative to the native sequence, fused to SIINFEKL. Of these peptides, only ANXA2-OVA containing the first N-terminal 36 amino acids induced TLR2-mediated signaling (Fig. 3a). Similarly, only immunization with ANXA2-OVA elicited significantly higher numbers of SIINFEKL/H-2K^b^ specific peripheral blood CD8^+^ T cells in B6 mice compared to ANXA2ΔΝ15-OVA or scrambled control (Fig. 3b).

## Conclusions

These studies leave open the question of ANXA2 as potential DAMP in vivo. ANXA2 expression is elevated in many tumors [3, 18], and in many cancers high levels of ANXA2 portend a poor prognosis [19]. These observations are consistent with ANXA2 signaling through TLR2, as TLR2-induced inflammation is implicated in liver and colon tumor metastasis, for example [20]. Our data suggest that immunogenic activity is absent or is overridden by other potentially tumor-promoting functions of ANXA2 [21–23], or by tumor-derived immune suppressive mechanisms such as regulatory T cells or myeloid-derived suppressor cells [24, 25]. It is also possible that ANXA2m is not present in great enough quantities, is sequestered, or exists in its tetrameric form that does not bind TLR2. The future may bring novel approaches to increase the immunogenicity of ANXA2 within tumor cells as a strategy to increase anti-tumor immunity in vivo.

## Methods

### Animal models

C57BL/6 J (B6), Tlr2-B6.129-Tlr2tm1Kir/J (*TLR2*^−/−^), and B6.B10ScN-*Tlr4*^*lps-del*^/JthJ (*TLR4*^*−/−*^) mice were purchased from Jackson Laboratories and maintained in specific pathogen-free facilities at the University of Minnesota. The murine GL261 glioma model was established as described [10, 16]. Mice were immunized with 50 μg OVA plus 50 μg of the indicated peptides four consecutive days, and once more, 7 days following the first vaccine [15]. All animal studies were reviewed and approved by the Institutional Animal Care and Use Committee at the University of Minnesota.

### Cell culture

GL261 cells were cultured in DMEM/F12 (1:1) with L-glutamine, sodium bicarbonate, penicillin/streptomycin (100 U/mL), B27 and N2 supplements, and 0.1-mg/mL Normocin under 5 % or 20 % O_2_ as described [10]. Splenocytes and T cells were cultured in RPMI 1640 containing 10 % heat-inactivated FBS, penicillin/streptomycin (100 U/ml), and 0.1 mg/ml Normocin.

### Immunoblot

GL261 cells were washed, pelleted, and lysed in radioimmunoprecipitation assay buffer containing protease and phosphatase inhibitors (Pierce). Lysates were diluted to the same protein concentration and were separated by 4–12 % SDS-PAGE. The separated proteins were then transferred to nitrocellulose, blocked, and incubated with mouse anti-ANXA2 (BD Biosciences). Blots were then washed and incubated with anti-mouse IgG HRP (Jackson ImmunoResearch). After additional washing, nitrocellulose was then incubated in ECL Plus chemiluminescent substrate (GE), drained, and exposed to HyBlot CL Autoradiography film (Denville Scientific).

### Immunoprecipitation

Cold GL261 lysate was adjusted to a concentration of 2 mg/ml. Primary Ab was added to 0.5 ml lysate followed by overnight incubation at 4 °C with rocking. Protein A/G Agarose (Santa Cruz) was added and incubated at 4 °C with rocking. Beads were then washed, resuspended in reducing sample buffer, supernatants separated by 4–12 % SDS-PAGE, and transferred to nitrocellulose as described above for immunoblotting. The Abs were mouse anti-mouse TLR2 (Clone T2.5, Santa Cruz), mouse anti-mouse ANXA2 (Clone 5 BD Biosciences), and anti-mouse IgG HRP (Jackson ImmunoResearch).

### Bone marrow-derived DC and TLR activity

For DC maturation experiments, B6 bone marrow-derived DCs (BMDCs) were generated using a modified protocol [26]. Briefly, femurs and tibias were removed and flushed with saline. Erythrocytes were lysed, and remaining leukocytes were washed and placed in 10 cm dishes at 2 × 10^6^ cells/10 ml in complete RPMI 1640 and 20 ng/ml GM-CSF (Peprotech). Non-adherent cells were removed and media were replaced every 3 days. Six days post-bone marrow harvest, loosely adherent cells were washed and plated in RPMI/GM-CSF.

HEK-Blue reporter cells stably express membrane-bound human TLR2 or human TLR4 and secrete alkaline phosphatase upon TLR stimulation (InvivoGen). HEK-Blue cells and BMDC swere stimulated with 50 ng/ml of the TLR2 agonist Pam_3_CSK_4_ (Imgenex), 2.5 μg/ml human ANXA2m (69 μM; AbD Serotec and Aviva Systems Biology), 6.5 μg/ml purified bovine ANXA2t (69 μM, Meridian), 5 μg/ml imiquimod, 2.6 μg/ml hu annexin A5 (69 μM, AbD Serotec), or 200 μg/ml ANXA2-OVA (STVHEILCKLSLEGDHSTPPSAYGSVKPYTNFDAEEQLESIINFEKLTEWT) peptides (residues 1–36 from ANXA2 followed by 16 OVA-derived aa). Control peptides, one containing the same content but scrambled order of the 36 ANXA2 aa (“Scrambled ANXA2-OVA,” GSCTESIEALHVLELVSPYTKSHNTPDSKGDYPFAEQLESIINFEKLTEWT), and one lacking the first 15 N terminal ANXA2 aa (“ANXA2ΔΝ15-OVA,” HSTPPSAYGSVKPYTNFDAEEQLESIINFEKLTEWT) were added to wells at levels equimolar to ANXA2-OVA. Forty-eight h following stimulation, BMDCs were stained and analyzed by flow cytometry, and HEK-Blue cell supernatant was assayed for secreted embryonic alkaline phosphatase activity using QuantiBlue colorimetric enzyme assay (InvivoGen). Enzymatic activity was recorded as an increase in OD640 after being normalized to media-only treatment.

### Cross-priming and cross-presentation

Splenocytes from B6, *TLR2*^−/−^, or *TLR4*^−/−^ mice were cultured at 2 × 10^5^ cells/well in a 96-well plate in complete RPMI 1640 media. Splenocytes were pulsed with combinations of the following: 10 μg tumor lysate, 2.5 μg/ml human ANXA2m (AbD Serotec), or 50 μg/ml hu gp100_25–33_ peptide (KVPRNQDWL) or an OVA-derived peptide (EVSQLEQLESIINFEKLTEEWTSSNVM). For measurement of cross-presented OVA peptide, splenocytes were harvested, stained for CD11c, MHCII, and SIINFEKL/H-2K^b^ complex, and analyzed by flow cytometry as described below. To measure IFN-γ, CD8^+^ T lymphocytes were purified from pmel-1 mouse [12] splenocytes (Miltenyi Biotec), and 2 × 10^5^ per well were co-cultured with unfractionated syngeneic splenocytes. Forty-eight hours after co-culture, IFN- γ was quantified in media using a flow cytometric bead array (BD Biosciences).

Cross-priming was tested in vivo through sequential daily vaccination as described [15, 27]. Briefly, B6 or *TLR2*^*−/−*^ mice were vaccinated four consecutive days with 100 μg OVA protein (with or without 1.5 μg human ANXA2m; AbD Serotec), 40 μg ANXA2-OVA peptide, or equimolar amounts of control peptides. Mice were boosted 3 days following the fourth vaccine, and bled the following day for analysis.

### Flow cytometry

Analyses of labeled cell suspensions were performed on a custom Canto II (BD) and analyzed with FlowJo software (Tree Star, Inc.). Fluorochrome-conjugated mAbs used were CD11c—FITC (eBioscience, clone N418), CD86—PE (eBioscience, clone GL1), SIINFEKL-H-2K^b^—PE (eBioscience, clone 25-D1.16) CD80—APC (eBioscience, clone 16-10A1), CD8α -APC (eBioscience, clone 53–6.7) and I-A/I-E—eFluor450 (MHC II; eBioscience, clone M5/114.15.2). The corresponding isotype controls were also purchased from eBioscience. A PE—dextran-conjugated multimer of SIINFEKL/H-2K^b^ was purchased from Immudex (Copenhagen, Denmark).

### CMV antigen cross-priming by human monocyte-derived DCs

5 × 10^5^ HLA-A2^+^ immature DCs were pulsed with 10 μg/ml of pp65_495–503_ (NLVPMVATV), with or without addition of 2.5 μg/ml human ANXA2m and matured. Purified donor-matched CD8 T cells were added and incubated as described previously [16].

## Abbreviations

ANXA2: annexin A2
ANXA2m: monomeric annexin A2
ANXA2t: tetrameric annexin A2
APC: antigen-presenting cell
B6: C57BL/6 J
BMDC: bone marrow-derived dendritic cell
CMV: cytomegalovirus
DAMP: danger-associated molecular pattern
DC: dendritic cell
MFI: median fluorescence intensity
N: amino
OVA: chicken ovalbumin
TLR: toll-like receptor
*TLR2*^−/−^: Tlr2-B6.129-Tlr2tm1Kir/J
*TLR4*^*−/−*^: B6.B10ScN-*Tlr4*^*lps-del*^/JthJ
